# Computational fluid dynamics simulations of blood flow regularized by 3D phase contrast MRI

**DOI:** 10.1186/s12938-015-0104-7

**Published:** 2015-11-26

**Authors:** Vinicius C. Rispoli, Jon F. Nielsen, Krishna S. Nayak, Joao L. A. Carvalho

**Affiliations:** Department of Electrical Engineering, University of Brasilia, Brasília, Brazil; UnB Gama College, University of Brasilia, Brasília, Brazil; fMRI Laboratory, Biomedical Engineering Department, University of Michigan, Ann Arbor, USA; Magnetic Resonance Engineering Laboratory, Ming Hsieh Department of Electrical Engineering, University of Southern California, Los Angeles, USA

**Keywords:** Computational fluid dynamics, Phase contrast, Magnetic resonance imaging

## Abstract

**Background:**

Phase contrast magnetic resonance imaging (PC-MRI) is used clinically for quantitative assessment of cardiovascular flow and function, as it is capable of providing directly-measured 3D velocity maps. Alternatively, vascular flow can be estimated from model-based computation fluid dynamics (CFD) calculations. CFD provides arbitrarily high resolution, but its accuracy hinges on model assumptions, while velocity fields measured with PC-MRI generally do not satisfy the equations of fluid dynamics, provide limited resolution, and suffer from partial volume effects. The purpose of this study is to develop a proof-of-concept numerical procedure for constructing a simulated flow field that is influenced by both direct PC-MRI measurements and a fluid physics model, thereby taking advantage of both the accuracy of PC-MRI and the high spatial resolution of CFD. The use of the proposed approach in regularizing 3D flow fields is evaluated.

**Methods:**

The proposed algorithm incorporates both a Newtonian fluid physics model and a linear PC-MRI signal model. The model equations are solved numerically using a modified CFD algorithm. The numerical solution corresponds to the optimal solution of a generalized Tikhonov regularization, which provides a flow field that satisfies the flow physics equations, while being close enough to the measured PC-MRI velocity profile. The feasibility of the proposed approach is demonstrated on data from the carotid bifurcation of one healthy volunteer, and also from a pulsatile carotid flow phantom.

**Results:**

The proposed solver produces flow fields that are in better agreement with direct PC-MRI measurements than CFD alone, and converges faster, while closely satisfying the fluid dynamics equations. For the implementation that provided the best results, the signal-to-error ratio (with respect to the PC-MRI measurements) in the phantom experiment was 6.56 dB higher than that of conventional CFD; in the in vivo experiment, it was 2.15 dB higher.

**Conclusions:**

The proposed approach allows partial or complete measurements to be incorporated into a modified CFD solver, for improving the accuracy of the resulting flow fields estimates. This can be used for reducing scan time, increasing the spatial resolution, and/or denoising the PC-MRI measurements.

## Background

Knowledge of blood flow patterns in the human body is a critical component in cardiovascular disease research and diagnosis. Two different approaches to 3D flow assessment are currently available to the researcher and clinician: direct, model-independent velocity mapping using phase contrast magnetic resonance imaging (PC-MRI) [1–3] or Doppler ultrasound, and model-based computational fluid dynamics (CFD) calculations [4–16]. Among the direct methods, PC-MRI has gained prominence in recent years due to its unrestricted 3D anatomical coverage and minimal operator dependence [1, 17–19]. The connection between MRI-based complex blood flow analysis (such as, turbulence [20] and helical blood flow [21]) and MRI-based biomarkers (such as, wall shear stress [22–24] and pressure gradients  [25–29]) with disease progression and diagnosis are active and promising areas of research. However, PC-MRI provides limited spatial and temporal resolutions, which inevitably impacts the accuracy of MRI-based hemodynamic parameter estimates [30].

CFD is an alternative that has been used to predict flow patterns in various vascular geometries, including intracranial aneurysms [10], the thoracic aorta [11], and the carotid bifurcation, both in models [12–15] and in vivo [16]. The equations describing Newtonian fluid flow are solved numerically for specified boundary and initial condition data. Such approach provides arbitrarily high spatial and temporal resolution, and is in principle capable of estimating flow fields for arbitrarily complex vessel geometries. Absolute hemodynamic parameter estimates can be obtained directly from the high-resolution flow fields produced by CFD, obviating the need for data smoothing or interpolation schemes.

The accuracy of conventional CFD routines hinges on many modeling assumptions that are not strictly true for in vivo vascular flow, including rigid vessel walls and uniform blood viscosity. Indeed, the proper choice of the underlying physics model is itself an open research question [10]. CFD predictions have so far shown variable agreement with PC-MRI measurements [10, 11, 13, 15], and the applicability of CFD to robust flow estimation is still being debated.

The use of fluid mechanics techniques for improving PC-MRI data is an active research field. Several algorithms from the literature use regularizations based on curl and divergence of the velocity field, which are associated with the irrotationality and incompressibility characteristics of the fluid flow, respectively. These include algorithms capable of improving streamlines [31, 32], and also algorithms for denoising the PC-MRI data [33–36].

However, the solutions found by those methods do not necessarily satisfy the Navier-Stokes momentum equation. Other authors integrated point-based measurements within a CFD solver, by adding a “force” term to the momentum equations that is proportional to the difference between predicted and measured velocities for a given grid point [37–39]. They used such an approach to integrate, respectively, Doppler-ultrasound velocity measurements and cerebral aneurysm blood flow MRI data into a CFD solver.

More recently, a method to accelerate 4D cardiac flow MRI using CFD simulations was proposed. The image model was generated by integrating numerical blood flow simulations (calculated using openFOAM [40]) into the MRI image-reconstruction algorithm [41]. However, this approach can not guarantee that the fluid physics model (momentum and continuity equations) is satisfied by the reconstructed velocity map.

Conventional CFD uses medical imaging data only to specify the vessel geometry and the flow at the inlet and outlet boundaries, or other previously known initial and boundary condition data, and uses the assumed fluid physics model to find the solution in the interior of the calculation domain [28]. The goal of this work is to develop a more general, flexible and easy to implement numerical framework for harnessing additional PC-MRI velocity measurements to construct a more robust and potentially more accurate CFD-based solution, considering PC-MRI data as ground truth. The proposed method is able to make use of full (or incomplete) PC-MRI measurements of one or more velocity components within the entire 3D volume. This is achieved through generalized Tikhonov regularization [42], obtaining a numerical solution that is close enough to the measured flow data; satisfies the fluid physics equations; reduces noise; and, in the clinical environment, can be used to reduce scan time.

Finally, this work is presented as a proof of concept of the CFD–MRI combined solver. All simulations herein were made using the finite volume method and SIMPLER algorithm in Cartesian grids, with unrealistic assumptions about the blood flow model, such as rigid walls and Newtonian viscosity. Nevertheless, the optimal numerical solution proposed in this work is general enough to be implemented: for any type of discretization method, such as finite differences, finite volume or finite element; for steady or unsteady flow; and, for any realistic physics model, such as non-Newtonian viscosity, elastic vessel walls, or slightly compressible flow. Even in the most realistic model, the discretization (finite differences, finite volume or finite elements) of the nonlinear set of differential equations produces a large and sparse system of linear equations, that forms the basis of the proposed numerical solution. The feasibility of the proposed approach is demonstrated on data from the carotid bifurcation of one healthy volunteer, and from a pulsatile carotid flow phantom. Two implementations of the regularized computational solution were evaluated and compared: one using only the PC-MRI data corresponding to the main velocity component (*z* axis); and another, using PC-MRI data corresponding to all three velocity components.

## Theory

### Blood flow model

The general model for fluid motion in 3D Euclidian space is given by the Navier–Stokes momentum equation [43]:

1 $$\begin{aligned} \rho \left( \frac{\partial \vec {\nu }}{\partial t}+\vec {\nu }\cdot \nabla \vec {\nu }\right) =-\nabla p-\nabla \cdot \hat{\tau }+\vec {b}, \end{aligned}$$

where $\rho$ is the fluid density, $\vec {\nu }=(u,v,w)$ is the flow velocity vector (*u*, *v*, and *w* are the velocity components associated with spatial axes *x*, *y*, and *z*, respectively), *t* is time, $\nabla$ is the gradient operator, *p* is the hydrodynamic pressure, $\hat{\tau }$ is the stress tensor, and $\vec {b}$ represents the body forces acting on the fluid during the flow. The stress tensor represents the momentum transferred in virtue of the molecular motions and interactions within the fluid. It is a function of the scalar invariants of the strain rate tensor $\hat{e}= (1/2) \left[ \nabla \vec {\nu }+(\nabla \vec {\nu })^{T} \right]$, where $^T$ denotes the transpose operation. For an incompressible fluid, it can be written as $\hat{\tau } = -\mu (\hat{e}) \, \hat{e}$, where the scalar $\mu (\hat{e})$ is the generalized Newtonian viscosity for a given $\hat{e}$.

In this work, blood is modeled as a Newtonian, incompressible and isothermal fluid, with constant viscosity $\mu$ and constant density $\rho$. We are also assuming that there are no body forces acting on the blood flow. Then, the simplification of the general momentum equation, Eq. (1), provides our blood model equation [43]:

2 $$\begin{aligned} \rho \left( \frac{\partial \vec {\nu }}{\partial t}+\vec {\nu }\cdot \nabla \vec {\nu }\right) =-\nabla p+\mu \Delta \vec {\nu }, \end{aligned}$$

where $\Delta$ is the Laplacian operator.

Since there are no sources of blood inside an artery, the flow field must also satisfy mass conservation [43], which is expressed by the continuity equation:

3 $$\begin{aligned} \nabla \cdot \vec {\nu }=0. \end{aligned}$$

### SIMPLER algorithm

Equations (2) and (3) must be solved for the unknown scalar field variables *u*, *v*, *w*, and *p*. Those equations are non-linear and coupled, and attempting to solve them directly in one step is a formidable, if not impossible, task.

The semi-implicit method for pressure-linked equations revised (SIMPLER) algorithm [44] is a well-known and established numerical routine for solving the momentum and continuity equations, subject to given boundary and initial conditions. It belongs to a class of algorithms capable of solving the non-linear coupled fluid dynamic equations, which also includes the SIMPLE, SIMPLEC, and PISO algorithms [45]. For our purposes, SIMPLER’s major advantage is that it does not require an initial guess for the pressure field; instead an initial estimate of the velocity field is used.

**Fig. 1:**
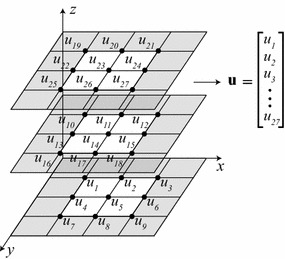
Discretization of the velocity component *u* on the computational grid, and representation as a stacked column vector

The discretization of the momentum equation, Eq. (2), forms the basis of the iterative CFD routine, yielding three linear systems. Let *N* be the total number of grid points in the discrete 3D calculation domain, i.e., in a rectangular grid, $N=N_{x}\cdot N_{y}\cdot N_{z}$, where $N_{x}$, $N_{y}$, and $N_{z}$ represent the number of grid points along the *x*, *y*, and *z* axes, respectively. Then, for the *n*-th iteration, we have:

4 $$\begin{aligned} {\mathbf {S}}_{u,n-1}\mathbf {u}_{n}&= {\mathbf {f}}_{u,n-1} \end{aligned}$$

5 $$\begin{aligned} {\mathbf {S}}_{v,n-1}{\mathbf {v}}_{n}& = {\mathbf {f}}_{v,n-1} \end{aligned}$$

6 $$\begin{aligned} {\mathbf {S}}_{w,n-1}{\mathbf {w}}_{n}&= {\mathbf {f}}_{w,n-1}, \end{aligned}$$

where ${\mathbf {S}}_{u,n-1}$, ${\mathbf {S}}_{v,n-1}$, and ${\mathbf {S}}_{w,n-1}$ are $N\times N$ square hepta-diagonal sparse matrices, each containing previous iteration information about all three velocity components, as well as the values of the density and viscosity constants; the three $N\times 1$ column vectors ${\mathbf {u}}_{n}$, ${\mathbf {v}}_{n}$, and ${\mathbf {w}}_{n}$ store the current iteration of the *u*, *v*, and *w* velocity component values, respectively, associated with all grid points in the 3D calculation domain (Fig. 1); and each of the constant $N\times 1$ column vectors ${\mathbf {f}}_{u,n-1}$, ${\mathbf {f}}_{v,n-1}$, and ${\mathbf {f}}_{w,n-1}$ contains previous iteration information about all three velocity components, current iteration pressure difference values, and the physical parameters $\rho$ and $\mu$.

The main difficulty when attempting to predict the flow of an incompressible fluid is that there is no equation for pressure. Hence, a discretized pressure equation is deduced applying Eqs. (4), (5), and (6) in the discretized continuity equation, giving rise to a discretization of the following Poisson equation:

7 $$\begin{aligned} \Delta p=\frac{1}{\delta t}\nabla \cdot \vec {\nu }, \end{aligned}$$

where $\delta t$ is the time step, i.e. the time increment between iterations (this is further discussed in the next section). For a given time instant $t=t_i$, convergence is achieved when $\nabla \cdot \vec {\nu }=0$. The pressure field is updated at each iteration based on the current velocity field estimate, and hence does not appear explicitly in Eqs. (4), (5), and (6). It is important to note that *u*, *v*, and *w* values must be defined on regular grids, staggered by half a grid spacing (along the three directions) with respect to the grid on which pressure values are defined. This is to avoid non-physical wiggle solutions for the pressure and velocity fields [44].

The steps of the SIMPLER algorithm, for a given time instant $t = t_i$, are summarized in Algorithm 1. A detailed explanation of the discretization of the Navier–Stokes equations is provided in Refs. [44, 45].
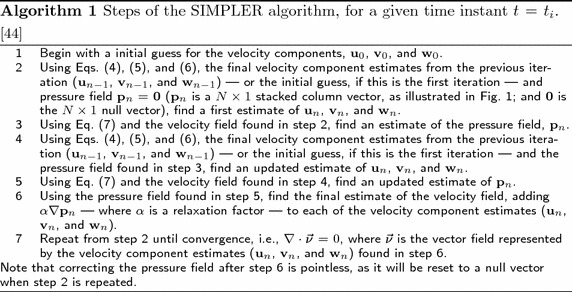


## Methods

### Algorithm implementation

On constructing the numerical solution of the unsteady Navier–Stokes equation, we assume that a velocity field and the boundary conditions at a given time instant $t = t_0$ are known. For this initial set of data, the numerical solution for the next time step $t_1=t_0+\delta t$ is constructed, and it converges toward the solution when the continuity equation is satisfied (step 7 in Algorithm 1). Starting with the solution for $t_1$, the same iterative procedure is repeated to obtain the solution for $t_2 = t_1 + \delta t$, and so forth. In this manner, a time-dependent flow field is computed.

In unsteady flow simulations of the Navier–Stokes equations by implicit numerical routines, the nature of the transient SIMPLER iterative procedures is equivalent to steady-state SIMPLER calculations applied, until convergence, for each time instant [45]. In other words, solving transient problems using SIMPLER is equivalent to solving successive steady-state problems. Moreover, steady-state calculations may be interpreted as pseudo-transient solutions with spatially-varying time steps [45]. In other words, the steady-state solutions are, in practice, unsteady solutions, considering a virtual time step $\delta t$ with fixed boundary conditions and initial data. This approach has been used by many recent authors [13, 16, 37–39]. While this was the numerical strategy adopted in this work, the approach proposed here can also be used for unsteady flow predictions.

The steady-state solution $\vec {\nu }_{\infty }$ corresponding to a given cardiac phase (a temporal frame within the cardiac cycle) is calculated using the MRI-measured inlet and outlet velocities for that cardiac phase. CFD calculations begin with an initial guess for $\vec {\nu }$, and simulations are carried forward in time until convergence, i.e.:

8 $$\begin{aligned} \frac{\left\| \vec {\nu }(t+\delta t)-\vec {\nu }(t)\right\| }{\delta t}<\varepsilon , \end{aligned}$$

given a suficiently small $\varepsilon$ ($\left\| \cdot \right\|$ denotes vector magnitude) and suficiently small time step $\delta t$. Note that, here, time *t* is a simulation-only parameter, and is unrelated to time instants within the cardiac cycle. It is also not related with the iteration steps (*n*) of the SIMPLER algorithm (Algorithm 1), since the entire algorithm—with multiple iterations until convergence criterion $\nabla \cdot \vec {\nu }=0$ is satisfied—is performed at each time instant *t*, until the convergence criterion shown in Eq. 8 is satisfied. At this point, $\vec {\nu }_{\infty }$ is obtained. If multiple cardiac phases were to be reconstructed, then $\vec {\nu }_{\infty }$ would be independently calculated for each cardiac phase.

Our implementation of the SIMPLER algorithm was validated with the bidimensional lid-driven cavity flow problem, known in the literature as a benchmark for testing CFD algorithms [46–48]. All algorithms were implemented in Matlab (The MathWorks, Inc., Natick, MA, USA). Linear systems were solved using the biconjugate gradients stabilized method.

### Proposed numerical solution

In this paper, we solve for a simulated velocity field, $\vec {\nu }_{\infty } = (u_{\infty },v_{\infty },w_{\infty })$, that is close enough to the MRI-measured vector field $\vec {\nu }_{\mathrm {mri}} = (u_{\mathrm {mri}},v_{\mathrm {mri}},w_{\mathrm {mri}})$, and satisfies the fluid dynamics equations, Eqs. (2) and (3).

Let *M* be the total number of voxels in the reconstructed $\vec {\nu }_{\mathrm {mri}}$ 3D velocity field, i.e., $M=M_{x}\cdot M_{y}\cdot M_{z}$, where $M_{x}$, $M_{y}$, and $M_{z}$ represent the number of voxels along the *x*, *y*, and *z* axes, respectively. Consider ${\mathbf {u}}_{\mathrm {mri}}$, ${\mathbf {v}}_{\mathrm {mri}}$, and ${\mathbf {w}}_{\mathrm {mri}}$ as the stacked $M \times 1$ column vectors with the PC-MRI measurements. Since the numerical solution of the Navier–Stokes continuity-equation system is based on the solution of linear systems, we propose that our numerical optimal solution is obtained by minimizing, for each velocity component, at iteration *n*, the following equations:

9 $$\begin{aligned} J_u({\mathbf {u}}_n)&= \frac{1}{2}||{\mathbf {S}}_{u}{\mathbf {u}}_{n}-{\mathbf {f}}_{u} ||^2 + \frac{\lambda _u}{2}||\Gamma _u{\mathbf {u}}_{n}-{\mathbf {u}}_\mathrm {mri} ||^2 \end{aligned}$$

10 $$\begin{aligned} J_v({\mathbf {v}}_n)&= \frac{1}{2}||{\mathbf {S}}_{v}{\mathbf {v}}_{n}-{\mathbf {f}}_{v} ||^2 + \frac{\lambda _v}{2}||\Gamma _v{\mathbf {v}}_{n}-{\mathbf {v}}_{\mathrm {mri}} ||^2 \end{aligned}$$

11 $$\begin{aligned} J_w({\mathbf {w}}_n)&=\frac{1}{2}||{\mathbf {S}}_{w}{\mathbf {w}}_{n}-{\mathbf {f}}_{w} ||^2 + \frac{\lambda _w}{2}||\Gamma _w{\mathbf {w}}_{n}-{\mathbf {w}}_{\mathrm {mri}} ||^2. \end{aligned}$$

The first term on the right hand side of Eqs. (9)–(11) is related to the numerical solution of the Navier–Stokes continuity equations, and the second term is related to the comparison between the numerical solution and the PC-MRI velocity field. The $\mathbf {S}$ matrices and $\mathbf {f}$ vectors are defined in Eqs. (4)–(6), but note that we dropped the “$n-1$” subscripts for simplicity and clarity; these are updated by velocity and pressure values calculated in the previous iteration. Coefficients $\lambda _u$, $\lambda _v$, and $\lambda _w$ are regularization factors, which weight consistance with PC-MRI data against conformance with the momentum equations. Matrices $\Gamma _u$, $\Gamma _v$, and $\Gamma _w$ are of size $M\times N$, and model the blurring effects due to finite k-space coverage in PC-MRI (this is further discussed below), while adjusting the number of points on the CFD grid in order to allow a comparison between $\vec {\nu }_n$ and $\vec {\nu }_\mathrm {mri}$. In this approach, the number of grid points in the CFD and MRI grids are not necessarily the same; we can use a finer grid in CFD than in MRI, for example. The optimal solutions for Eqs. (9)–(11) are straightforward [42], and given by

12 $$\begin{aligned} {\mathbf {u}}_n&= \left( {\mathbf {S}}_{u}^T {\mathbf {S}}_{u} +\lambda _u\Gamma _u^T \Gamma _u\right) ^{-1}\left( {\mathbf {S}}_{u}^T{\mathbf {f}}_{u} +\lambda _u\Gamma _u^T{\mathbf {u}}_{\mathrm {mri}}\right) \end{aligned}$$

13 $$\begin{aligned} {\mathbf {v}}_n&= \left( {\mathbf {S}}_{v}^T {\mathbf {S}}_{v} +\lambda _v\Gamma _v^T \Gamma _v\right) ^{-1}\left( {\mathbf {S}}_{v}^T{\mathbf {f}}_{v} +\lambda _v\Gamma _v^T{\mathbf {v}}_{\mathrm {mri}}\right) \end{aligned}$$

14 $$\begin{aligned} {\mathbf {w}}_n&=\left( {\mathbf {S}}_{w}^T {\mathbf {S}}_{w} +\lambda _w\Gamma _w^T \Gamma _w\right) ^{-1}\left( {\mathbf {S}}_{w}^T{\mathbf {f}}_{w} +\lambda _w\Gamma _w^T{\mathbf {w}}_{\mathrm {mri}}\right) . \end{aligned}$$

To understand the construction of the $\Gamma$ matrices, consider that in the absence of noise, artifacts, and distortions, the MRI-measured vector field $\vec {\nu }_{\mathrm {mri}} = (u_{\mathrm {mri}},v_{\mathrm {mri}},w_{\mathrm {mri}})$ is a blurred version of the true vector field $\vec {\nu } = (u,v,w)$. For the *u* component, for example, we can write:

15 $$\begin{aligned} u_{\mathrm {mri}}(x,y,z) = u(x,y,z) *\psi _u(x,y,z), \end{aligned}$$

where $*$ denotes convolution, and blurring kernel $\psi _u(x,y,z)$ is the point-spread function associated with the k-space coverage that was used when measuring $u_{\mathrm {mri}}$. Similarly, we can write $v_{\mathrm {mri}} = v *\psi _v$ and $w_{\mathrm {mri}} = w *\psi _w$. If all three PC-MRI velocity components are measured using the same k-space coverage, then $\psi _u = \psi _v = \psi _w$. For a 3DFT acquisition, these spatial blurring kernels are equal to

16 $$\begin{aligned} \psi (x,y,z) = {\mathrm {sinc}}\left( \frac{x}{\delta x} \right) \times {\mathrm {sinc}}\left( \frac{y}{\delta y} \right) \times {\mathrm {sinc}}\left( \frac{z}{\delta z} \right) , \end{aligned}$$

where $\delta x$, $\delta y$ and $\delta z$ are the spatial resolutions of $\vec {\nu }_{\mathrm {mri}}$ along the *x*, *y*, and *z* axis, respectively.

We want the CFD-estimated vector field $\vec {\nu }_{\infty } = (u_{\infty },v_{\infty },w_{\infty })$ to be an accurate representation of the true vector field $\vec {\nu }$. If this is so, then we should expect $u_{\mathrm {mri}} \approx u_{\infty } *\psi _u$, $v_{\mathrm {mri}} \approx v_{\infty } *\psi _v$, and $w_{\mathrm {mri}} \approx w_{\infty } *\psi _w$. The discretization of these equations yields three linear systems. Then, using the same notation introduced earlier, for the *n*th iteration of the CFD algorithm, we can write:

17 $$\begin{aligned} {\mathbf {u}}_{\mathrm {mri}}&\approx \Gamma _u{\mathbf {u}}_{n} \end{aligned}$$

18 $$\begin{aligned} {\mathbf {v}}_{\mathrm {mri}}&\approx \Gamma _v{\mathbf {v}}_{n} \end{aligned}$$

19 $$\begin{aligned} {\mathbf {w}}_{\mathrm {mri}}& \approx \Gamma _w{\mathbf {w}}_{n} . \end{aligned}$$

The coefficients of $\Gamma _u$, $\Gamma _v$, and $\Gamma _w$ are calculated from $\psi _u$, $\psi _v$, and $\psi _w$, respectively. If all three PC-MRI velocity components are measured using the same k-space coverage, and reconstructed onto identical grids, then $\Gamma _u = \Gamma _v = \Gamma _w$.

The MRI-guided CFD estimate corresponding to one cardiac phase was calculated as a steady-state solution $\vec {\nu }_{\infty }$. All three components of the PC-MRI velocity field $\vec {\nu }_\mathrm {mri}$ measured at the *z* positions at the boundaries of the calculation domain were used as inlet and outlet boundary conditions for that cardiac phase. Note that this steady state solution $\vec {\nu }_{\infty }$ is the closest fit in the least-squares sense to the direct PC-MRI measurements that satisfy both momentum equation (Eq. 2) and continuity equation (Eq. 3). This is guaranteed by the fact that the optimal solutions Eqs. (12)–(14) are solved in each iteration of the SIMPLER algorithm (steps 2 and 4, in Algorithm 1), and by the convergence criterion (step 7).

In each of our experiments, all three PC-MRI velocity components were measured using the same k-space coverage, and reconstructed onto identical grids. In the phantom experiments, we used the same grid size for both $\vec {\nu }_{\mathrm {mri}}$ and $\vec {\nu }_{\infty }$, because the phantom data were measured with high spatial resolution. In these experiments, $\vec {\nu }_{\mathrm {mri}}$ was reconstructed without zero-padding, i.e., onto $\delta x \times \delta y \times \delta z$ voxels, and the CFD grid points were defined at the center of each of $\vec {\nu }_{\mathrm {mri}}$’s voxels. Hence, $\Gamma _u = \Gamma _v = \Gamma _w$ was defined as an identity matrix. In the in vivo experiments, $\vec {\nu }_{\mathrm {mri}}$ was reconstructed using 2-fold zero-padding along each of the spatial axes, since the data was acquired with low spatial resolution. Then, $\Gamma$ was an $N\times N$ symmetric matrix, with coefficients calculated from the point spread function $\psi (x,y,z)$, defined in Eq. (16). This infinite support point spread function was truncated by multiplication with the box function

20 $$\begin{aligned} B(x,y,z)=\text{ rect }\left( \frac{x}{2\delta x}\right) \times \text{ rect }\left( \frac{y}{2\delta y}\right) \times \text{ rect }\left( \frac{z}{2\delta z}\right) , \end{aligned}$$

where $\text{ rect }(w)=1$, if $-1\le w\le 1$, and $\text{ rect }(w)=0$, otherwise.

### Experimental setup: phantom demonstration

**Fig. 2:**
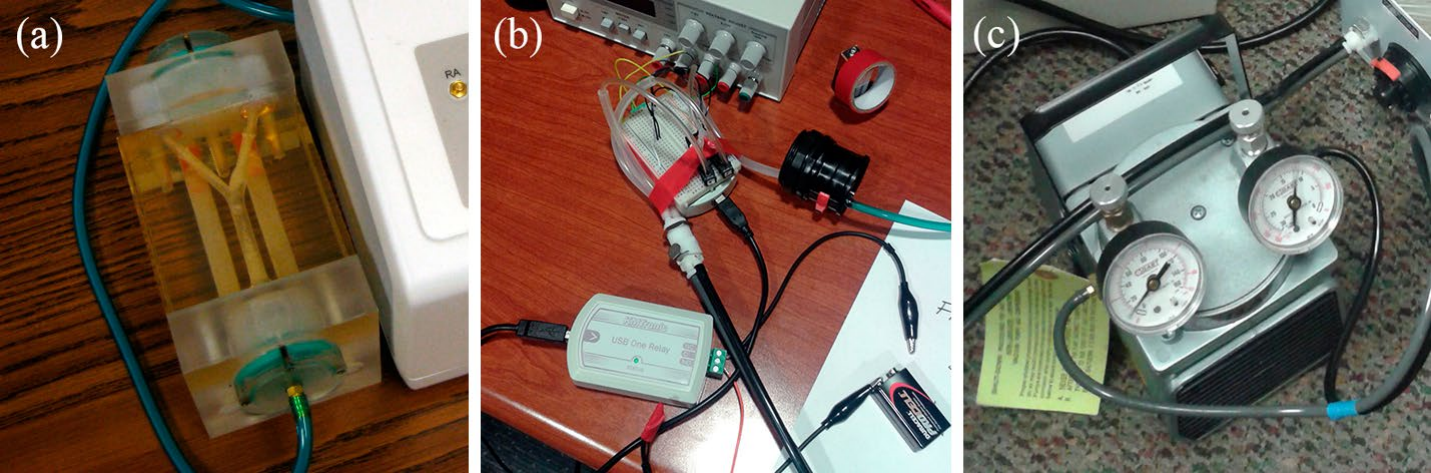
**a** Pulsatile carotid flow phantom (Phantoms by Design, Inc., Bothell, WA, USA) used to validate the proposed method; **b** pump controller that regulates flow frequency and gating signal; **c** air pump that generates the flow inside the phantom

PC-MRI data of a pulsatile carotid flow phantom (Phantoms by Design, Inc., Bothell, WA) (Fig. 2) were obtained with high spatial resolution and high signal–to–noise ratio, from four time-resolved 3DFT FGRE image volumes (three acquired each with a velocity encoding bipolar gradient on one of the three axes, and one without a bipolar gradient). The scan parameters were: $0.5\times 0.5\times 1.0$ mm$^3$ spatial resolution; FOV $4.0\times 3.5\times 5.0$ cm$^3$; TR 11.4 ms; flip angle 8.5$^\circ$; temporal resolution 91.2 ms; VENC 50 cm/s; 40 min per scan; 9 NEX. The data were acquired on a GE Discovery MR750 3T system (50 mT/m and 200 T/m/s max gradient amplitude and slew rate), with a 32-channel receive-only head coil array (Nova Medical, Inc., Wilmington, MA, USA). The through-slab (*z*) axis was oriented along the S/I direction. The phantom’s pulse cycle was set to 60 bpm.

Only the temporal frame corresponding to peak flow was reconstructed. PC-MRI velocity component maps $$u_{\mathrm {mri}},$$$$v_{\mathrm {mri}}$$ and $$w_{\mathrm {mri}}$$ were calculated using data from all channels of the receive coil array. The lumen was segmented by manually outlining the vessel borders from a stack of 2D axial images, obtained from the reconstructed 3D volume. A few voxels presented phase-wrap artifacts; these voxels were manually identified and their velocities were corrected by adding $$2\pi$$ to their values.

The combined solver calculations assumed fluid viscosity of $\mu$ = 0.005 Pa s and density of $\rho$ = 1100 kg/m$^3$ (these values were provided by the phantom manufacturer). Calculations were performed with time step $\delta t$ = 0.1 ms on a Cartesian grid of $0.5\times 0.5 \times 1.0$ mm$^3$ voxel size.

The CFD simulation domain was rectangular, of size $32.5\times 9.0\times 41.0\,\,\mathrm {mm}^{3}$. Each iteration required about 10 seconds of computation time on an Intel Core i7 processor running at 2.8 GHz.

Three simulated steady-state velocity fields $\vec {\nu }_{\infty }$ were obtained:

1. using the conventional SIMPLER algorithm, i.e., not using the PC-MRI data to constrain the CFD solution ($\vec {\nu }_\mathrm {mri}$ was used only as inlet and outlet velocities for the geometry);
2. using the velocity component associated with the main flow axis (*z*) measured with PC-MRI ($w_{\mathrm {mri}}$) to constrain the CFD solution (*u* and *v* components were determined solely from the fluid physics model); and
3. using all three velocity components measured with PC-MRI ($u_{\mathrm {mri}}$, $v_{\mathrm {mri}}$, and $w_{\mathrm {mri}}$) to constrain the CFD solution.

The first approach is equivalent to making $\lambda _w=\lambda _u=\lambda _v=0$; in the second approach, we used $\lambda _w=1$ and $\lambda _u=\lambda _v=0$; in the third approach, we used $\lambda _u=\lambda _v=\lambda _w=1$. All three approaches used all three components of the PC-MRI velocity field $\vec {\nu }_\mathrm {mri}$ measured at the *z* positions at the boundaries of the calculation domain as inlet and outlet boundary conditions. The number of iterations until convergence for the above simulations was 89, 40 and 5 iterations, respectively.

### Experimental setup: in vivo demonstration

PC-MRI data of the carotid bifurcation of one healthy volunteer were obtained from four time-resolved 3DFT FGRE image volumes (three acquired each with a velocity encoding bipolar gradient on one of the three axes, and one without a bipolar gradient). The scan parameters were: $1.0\times 1.0\times 2.5$ mm$^3$ spatial resolution; FOV $7.5\times 12.0\times 36.0$ cm$^3$; TR 7.0 ms; flip angle 15$^\circ$; temporal resolution 56 ms; VENC 160 cm/s; 7 min per scan; 1 NEX. The data were acquired on a GE Signa 3T EXCITE HD system (40 mT/m and 150 T/m/s max gradient amplitude and slew rate), with a 4-channel neck receive coil array. The through-slab (*z*) axis was oriented along the S/I direction. The institutional review board of the University of Southern California approved the imaging protocols. The subject was screened for MRI risk factors and provided informed consent in accordance with institutional policy.

Only the cardiac phase corresponding to peak flow was reconstructed. PC-MRI velocity component maps $$u_{\mathrm {mri}},$$$$v_{\mathrm {mri}}$$ and $$w_{\mathrm {mri}}$$ were calculated using data from only one channel of the receive coil array. Residual linear velocity offsets in each velocity component map (e.g., due to eddy-currents) were removed by performing a linear fit to manually defined 3D regions containing only stationary tissue. The lumen was segmented by manually outlining the vessel borders from a stack of 2D axial images, obtained from the reconstructed 3D volume.

The combined solver calculations assumed blood viscosity $\mu$ = 0.0032 Pa s and density of $\rho$ = 1060 kg/m$^3$ [49]. Calculations were performed with time step $\delta t$ = 0.25 ms on a Cartesian grid of $0.50\times 0.50 \times 1.25$ mm$^3$ voxel size. The CFD simulation domain was rectangular, of size $30\times 37\times 125$ mm$^3$ (the PC-MRI data was cropped to match this grid size). Each iteration required about 180 s of computation time on an Intel Core i7 processor running at 2.8 GHz.

Three simulated steady-state velocity fields $\vec {\nu }_{\infty }$ were obtained, using the same three approaches used in the phantom experiment. The number of iterations until convergence for the simulations was 1058, 190 and 6, respectively.

### Quantitative evaluation

The CFD-simulated velocity fields were quantitatively compared with the PC-MRI measurements by means of the signal-to-error ratio (SER). The SER measures the ratio between the energy of the signal and the energy of the estimation error. We considered the PC-MRI velocity field, $\vec {\nu }_{\mathrm {mri}} = (u_{\mathrm {mri}},v_{\mathrm {mri}},w_{\mathrm {mri}})$, as our ground-truth “signal”; consequently, the estimation error is the vector difference between the CFD-estimated velocity field, $\vec {\nu }_{\infty } = (u_{\infty },v_{\infty },w_{\infty })$, and the ground-truth field, $\vec {\nu }_{\mathrm {mri}}$. Thus, the SER is calculated (in decibels) as:

21 $$\begin{aligned}&{\mathrm {SER}}_{\vec {\nu }}=10\log _{10}\left( \frac{\sum _{i,j,k}\left\| \vec {\nu }_{\mathrm {mri}}(i,j,k)\right\| ^{2}}{\sum _{i,j,k} \left\| \vec {\nu }_{\infty }(i,j,k) - \vec {\nu }_{\mathrm {mri}}(i,j,k) \right\| ^{2}}\right) ,&\end{aligned}$$

where integers *i*, *j*, and *k* represent grid-point indexes along the *x*, *y*, and *z* axes, respectively. Similarly, the SER was also calculated individually for each of the velocity components, as:

22 $$\begin{aligned}&{\mathrm {SER}}_{u} = 10\log _{10}\left( \frac{\sum _{i,j,k} u_{\mathrm {mri}}(i,j,k)^2}{\sum _{i,j,k} \left[ u_{\infty }(i,j,k) - u_{\mathrm {mri}}(i,j,k)\right] ^{2}}\right)&\end{aligned}$$

23 $$\begin{aligned}&{\mathrm {SER}}_{v} = 10\log _{10}\left( \frac{\sum _{i,j,k} v_{\mathrm {mri}}(i,j,k)^2}{\sum _{i,j,k} \left[ v_{\infty }(i,j,k) - v_{\mathrm {mri}}(i,j,k)\right] ^{2}}\right)&\end{aligned}$$

24 $$\begin{aligned}&{\mathrm {SER}}_{w} = 10\log _{10}\left( \frac{\sum _{i,j,k} w_{\mathrm {mri}}(i,j,k)^2}{\sum _{i,j,k} \left[ w_{\infty }(i,j,k) - w_{\mathrm {mri}}(i,j,k) \right] ^{2}}\right) .&\end{aligned}$$

Using these SER values, the three CFD approaches—pure CFD, CFD driven by one PC-MRI velocity component, and CFD driven by all three PC-MRI velocity components—were quantitatively evaluated and compared.

### Evaluation of denoising properties

Under our hypothesis, CFD simulations provide a smooth, noise-free flow field. Therefore, we expect that the proposed approach can be used as a denoising mechanism for PC-MRI flow assessment. In order to verify the denoising effects of the combined solver, we added zero-mean Gaussian noise with standard deviation 8 cm/s to the phantom’s measured velocity field, $\vec {\nu }_{\mathrm {mri}}$.

This noisy flow field was used to constrain the CFD calculations, using the approach in which all three velocity components measured with PC-MRI are used. In this experiment, we used $\lambda _u=\lambda _v=\lambda _w=\lambda$; and four different values of $\lambda$ were evaluated: $5\times 10^{-9}$, $5\times 10^{-8}$, $5\times 10^{-7}$, and $5\times 10^{-6}$. The SER between the proposed approach and the original PC-MRI measurements was calculated, and compared with the SER of the noisy flow field. The pure CFD approach, in which the noisy PC-MRI data are used only as inlet and outlet velocities for the geometry, was also evaluated (this is equivalent to making $\lambda = 0$).

The phantom data was used in this denoising experiment, because it was acquired using 9 NEX—which results in high signal-to-noise ratio (SNR), while the in vivo data was acquired using only 1 NEX. The noise levels on the phantom’s measured velocity components—estimated as the standard deviation in regions of uniform mean velocity—are lower than 3 cm/s; while the SNR of the magnitude images exceeds 26 dB. The velocity-to-noise ratio (VNR) [50, 51] for the *u* and *w* components reach 28 and 31 dB, respectively (the VNR for the *v* component was not calculated, because *v* is approximately null over the entire geometry).

Finally, in order to justify our denoising experiment, we analyze the noise distribution in PC-MRI images. We note that, from a maximum likelihood perspective, Eqs. (9)–(11) assume that the PC-MRI data is degraded by Gaussian noise. Under certain conditions, one can prove that velocity field noise in PC-MRI satisfies a zero-mean Gaussian distribution [52]. Therefore, the additive noise acting on the velocity fields can be assumed to be Gaussian distributed [27, 52]. Hence, the proposed minimization is well-suited in terms of the MR noise distribution.

## Results

### Phantom demonstration

**Fig. 3:**
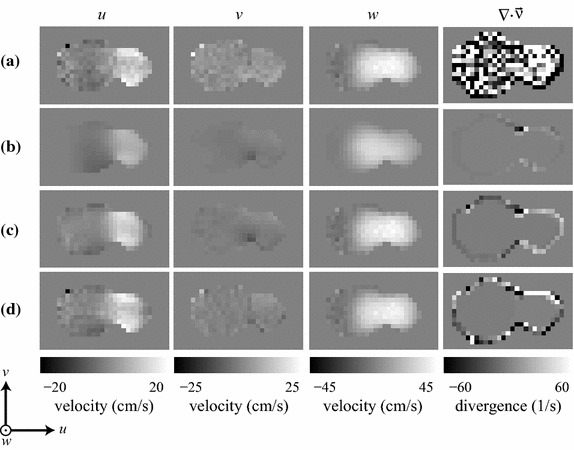
Velocity maps for the individual velocity components (*u*, *v*, and *w*) and divergence map of the velocity field ($\vec {\nu }$), for an axial slice at the bifurcation of the carotid flow phantom: **a** PC-MRI; **b** CFD; **c** CFD guided by PC-MRI data corresponding to the main velocity component ($w_{\mathrm {mri}}$); and **d** CFD guided by PC-MRI data corresponding to all three velocity components ($u_{\mathrm {mri}}$, $v_{\mathrm {mri}}$, and $w_{\mathrm {mri}}$)

**Fig. 4:**
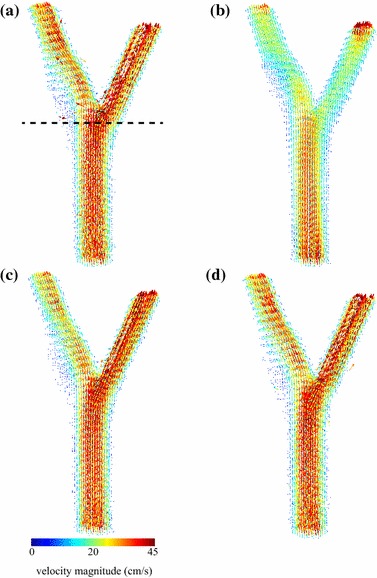
Vector field visualization of the velocity field ($\vec {\nu }$) over the entire tridimensional volume of the carotid flow phantom: **a** PC-MRI; **b** CFD; **c** CFD guided by PC-MRI data corresponding to the main velocity component ($w_{\mathrm {mri}}$); and **d** CFD guided by PC-MRI data corresponding to all three velocity components ($u_{\mathrm {mri}}$, $v_{\mathrm {mri}}$, and $w_{\mathrm {mri}}$). The *dotted line* in **a** indicates the position of the slice shown in Figs. 3 and 5

Figure 3 shows a qualitative velocity-map comparison between the PC-MRI phantom measurements and the three simulated results. The PC-MRI velocity field does not satisfy the continuity equation, since its divergence is nonzero within the lumen (Fig. 3a). The pure CFD solution produced a velocity field that satisfies the physical model, but is considerably smoother than the PC-MRI measurements (Fig. 3b). Using one MRI-measured velocity component ($w_{\mathrm {mri}}$) to guide the CFD simulation resulted in a solution that is qualitatively more similar to the MRI-measured field, while still satisfying the continuity and momentum equations (Fig. 3c). Even better agreement was achieved when all three MRI-measured velocity components ($u_{\mathrm {mri}}$, $v_{\mathrm {mri}}$, and $w_{\mathrm {mri}}$) were used to guide the CFD simulation (Fig. 3d). These improvements can be also appreciated on a vector field visualization of the flow field over the entire tridimensional volume (Fig. 4).

**Table 1:** Signal-to-error ratio (in dB) between the phantom experiment results from each of the three CFD approaches—pure CFD (labeled “CFD”); CFD driven by one PC-MRI velocity component (labeled “CFD + 1D”); and CFD driven by all three PC-MRI velocity components (labeled “CFD + 3D”), relative to the MRI-measured velocity field

	CFD	CFD + 1D	CFD + 3D
${\mathrm {SER}}_{u}$	2.97	4.16 ($\uparrow$)	6.74 ($\uparrow$)
${\mathrm {SER}}_{v}$	−0.25	$-$0.30 ($\approx$)	2.03 ($\uparrow$)
${\mathrm {SER}}_{w}$	5.44	16.53 ($\uparrow \uparrow$)	13.46 ($\uparrow$)
${\mathrm {SER}}_{\vec {\nu }}$	6.57	8.38 ($\uparrow$)	13.13 ($\uparrow \uparrow$)

SER values were calculated according to Eqs. (21)–(24)

Table 1 shows the SER between the phantom experiment results from each of the three CFD approaches, relative to the MRI-measured velocity field. The approach using only one PC-MRI velocity component ($w_{\mathrm {mri}}$) to drive the CFD calculations (labeled “CFD + 1D”) provided a 11.09 dB improvement in SER relative to pure CFD (labeled simply “CFD”) with respect to the *w* component; however, the improvement was only 1.81 dB when considering all three components. The approach using all three PC-MRI velocity components ($u_{\mathrm {mri}}$, $v_{\mathrm {mri}}$, and $w_{\mathrm {mri}}$) to drive the CFD calculations (labeled “CFD + 3D”) provided a 6.56 dB improvement in SER relative to pure CFD when considering all three components, while still providing a 8.02 dB improvement when considering only the *w* component.

Note that, for each of the CFD approaches, the SER was lower for the *u* and *v* components than it was for *w* (Table 1). This can be explained by the fact that the same VENC was used for measuring all three PC-MRI velocity components (which implies similar noise levels), but the velocities along the *z* axis are considerably higher than those along the *x* and *y* axes (the energy of $w_{\mathrm {mri}}$ was 11.42 dB higher than that of $u_{\mathrm {mri}}$, and 15.72 dB higher than that of $v_{\mathrm {mri}}$). As a consequence, the SNR of $w_{\mathrm {mri}}$ is substantially higher than that of $u_{\mathrm {mri}}$ and $v_{\mathrm {mri}}$. Thus, even if the energy of the absolute error between the CFD-estimated velocities and the MRI-measured velocities was the same for the three components, ${\mathrm {SER}}_{w}$ would be higher than ${\mathrm {SER}}_{u}$ and ${\mathrm {SER}}_{v}$. Also, note that CFD approaches provide a smooth, (ideally) noise-free velocity field, but the SER was calculated with respect to a noisy PC-MRI field. This means that the denoising properties of the CFD approaches could actually hurt the SER, especially if noise levels are relatively high when compared to the velocity values (this is the case for the *u* and *v* components). However, denoising is a desirable feature, so this does not necessarily indicate an unwanted result.

**Fig. 5:**
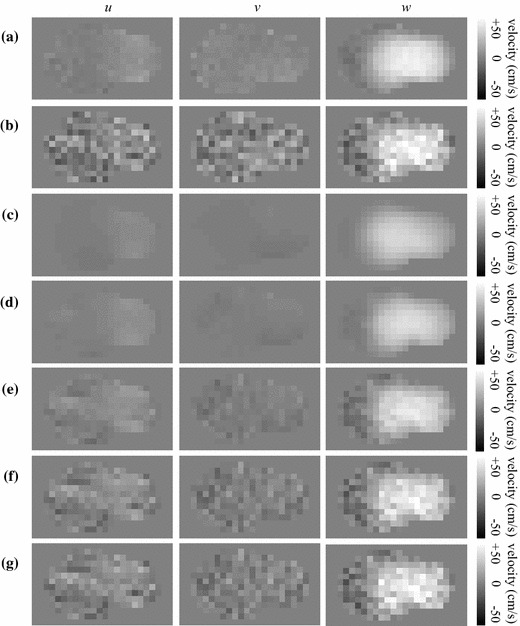
Velocity maps for the individual velocity components (*u*, *v*, and *w*), for an axial slice at the bifurcation of the carotid flow phantom: **a** PC-MRI; **b** PC-MRI with added Gaussian noise ($\sigma =8$ cm/s); **c** pure CFD solution using noisy PC-MRI data as inlet and oulet velocities; **d** CFD guided by the noisy PC-MRI data, with $\lambda =5\times 10^{-9}$; **e** CFD guided by the noisy PC-MRI data, with $\lambda =5\times 10^{-8}$; **f** CFD guided by the noisy PC-MRI data, with $\lambda =5\times 10^{-7}$; **g** CFD guided by the noisy PC-MRI data, with $\lambda =5\times 10^{-6}$

Figure 5 and Table 2 show the results of the experiment in which the denoising properties of the proposed approach were evaluated. CFD calculations were constrained by all three PC-MRI velocity components, with added Gaussian noise. The combined solver improved the SER of each individual velocity component, for all different weight parameters we evaluated (Table 2). The CFD solution constrained by PC-MRI using $\lambda =5\times 10^{-8}$ (Fig. 5e) provides a velocity field that is less noisy and visually more similar to the measured PC-MRI velocity field (Fig. 5a) than the pure CFD solution obtained using the noisy PC-MRI velocity field as boundary data (Fig. 5c). Using smaller values of $\lambda$ (Fig. 5d) results in solutions that are closer to the pure CFD solution (Fig. 5c). Using larger values of $\lambda$ (Figs. 5f, g) results in solutions that are closer to the noisy MRI data used to constrain the solution (Fig. 5b). These results can be appreciated quantitatively on Table 2. Relative to the noisy PC-MRI velocity field, the overall ($\vec {\nu }$) SER gain was 1.23 dB for $\lambda = 5\times 10^{-9}$; 2.47 dB for $\lambda = 5\times 10^{-8}$; 2.14 dB for $\lambda = 5\times 10^{-7}$; and 1.23 dB for $\lambda = 5\times 10^{-6}$. Moreover, all constrained CFD solutions presented better quantitative results than pure CFD. The overall ($\vec {\nu }$) SER gain, relative to pure CFD, was 0.55 dB for $\lambda = 5\times 10^{-9}$; 1.79 dB for $\lambda = 5\times 10^{-8}$; 1.46 dB for $\lambda = 5\times 10^{-7}$; and 0.55 dB for $\lambda = 5\times 10^{-6}$.

**Table 2:** Signal-to-error ratio (in dB) between noisy and original PC-MRI measurements; and between the MRI-guided CFD estimates and the original PC-MRI measurements

	Noisy	CFD	CFD+3D	CFD+3D	CFD+3D	CFD+3D
	PC-MRI	${\lambda =0}$	${\lambda =5\times 10^{-9}}$	${\lambda =5\times 10^{-8}}$	${\lambda =5\times 10^{-7}}$	${\lambda =5\times 10^{-6}}$
${\mathrm {SER}}_{u}$	0.80	2.66	2.87	3.22	2.34	1.75
${\mathrm {SER}}_{v}$	−2.46	−0.50	−0.41	−0.39	−1.18	−1.67
${\mathrm {SER}}_{w}$	4.64	5.03	5.82	7.20	5.88	5.06
${\mathrm {SER}}_{\vec {\nu }}$	5.71	6.39	6.94	8.18	7.85	6.94

Additive zero-mean Gaussian noise with standard deviation of $\sigma =8$ cm/s was used. CFD estimates were obtained using the combined solver with different values of the weight parameter $\lambda$, constrained by the noisy PC-MRI measurements. All three PC-MRI velocity components were used to guide the CFD calculations. SER values were calculated according to Eqs. (21)–(24)

These results illustrate the potential of the proposed numerical framework also as a denoising technique for PC-MRI.

### In vivo demonstration

**Fig. 6:**
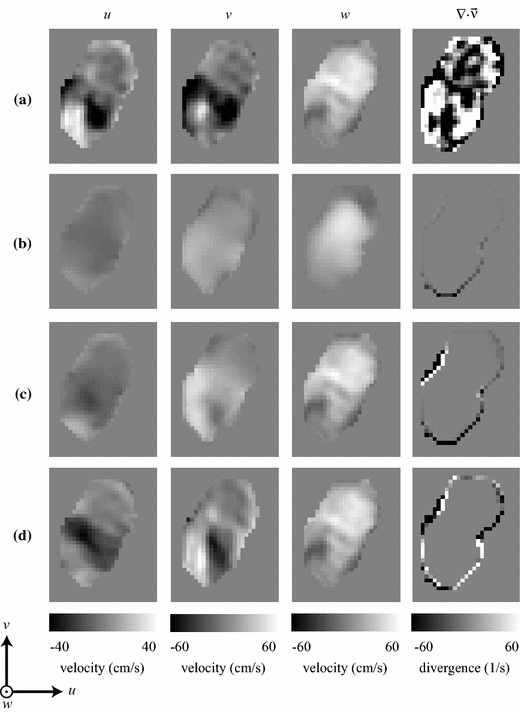
Velocity maps for the individual velocity components (*u*, *v*, and *w*) and divergence map of the velocity field ($\vec {\nu }$), for a slice perpendicular to the carotid bifurcation of a healthy volunteer: **a** PC-MRI; **b** CFD; **c** CFD guided by PC-MRI data corresponding to the main velocity component ($w_{\mathrm {mri}}$); and **d** CFD guided by PC-MRI data corresponding to all three velocity components ($u_{\mathrm {mri}}$, $v_{\mathrm {mri}}$, and $w_{\mathrm {mri}}$)

**Fig. 7:**
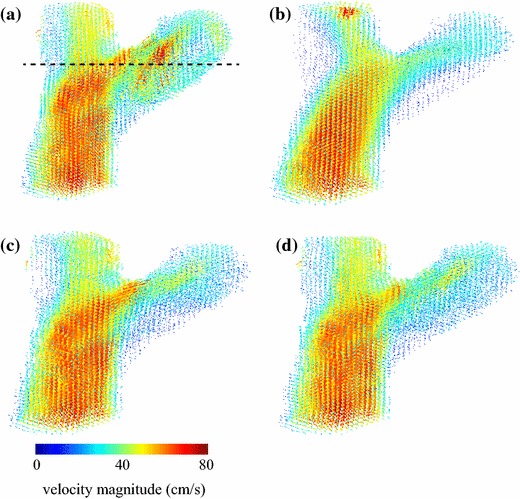
Vector field visualization of the velocity field ($\vec {\nu }$) over the entire tridimensional volume of the carotid bifurcation of a healthy volunteer: **a** PC-MRI; **b** CFD; **c** CFD guided by PC-MRI data corresponding to the main velocity component ($w_{\mathrm {mri}}$); and **d** CFD guided by PC-MRI data corresponding to all three velocity components ($u_{\mathrm {mri}}$, $v_{\mathrm {mri}}$, and $w_{\mathrm {mri}}$). The *dotted line* in **a** indicates the position of the slice shown in Fig. 6

Figure 6 provides a qualitative velocity-map comparison between the PC-MRI in vivo measurements and the three simulated results. As in the phantom experiment, the PC-MRI velocity field does not satisfy the continuity equation, since its divergence is nonzero within the lumen (Fig. 6a). The pure CFD solution produced a velocity field that satisfies the physical model, but differs considerably from the PC-MRI measurements (Fig. 6b). Using one MRI-measured velocity component ($w_{\mathrm {mri}}$) to guide the CFD simulation resulted in a solution that is qualitatively more similar to the MRI-measured field, while still satisfying the continuity and momentum equations (Fig. 6c). Even better agreement was achieved when all three MRI-measured velocity components ($u_{\mathrm {mri}}$, $v_{\mathrm {mri}}$, and $w_{\mathrm {mri}}$) were used to guide the CFD simulation (Fig. 6d). These improvements can be also appreciated on a vector field visualization of the flow field over the entire tridimensional volume (Fig. 7).

Table 3 shows the SER between the in vivo experiment results from each of the three CFD approaches, relative to the MRI-measured velocity field. The approach using only one PC-MRI velocity component ($w_{\mathrm {mri}}$) to drive the CFD calculations (labeled “CFD + 1D”) provided a 9.79 dB improvement in SER relative to pure CFD (labeled simply “CFD”) with respect to the *w* component; however, the SER was only 0.76 dB higher when considering all three components, since the agreement with PC-MRI for the *v* component was made worst. The approach using all three PC-MRI velocity components ($u_{\mathrm {mri}}$, $v_{\mathrm {mri}}$, and $w_{\mathrm {mri}}$) to drive the CFD calculations (labeled “CFD + 3D”) improved the SER for all three components, by 0.24, 0.59, and 6.00 dB, respectively; as a result, there was a 2.15 dB overall improvement in SER relative to pure CFD, and a 1.39 dB improvement relative to CFD guided only by $w_{\mathrm {mri}}$. As in the phantom experiments, the SER was lower for the *u* and *v* components than it was for *w*, for all three CFD approaches. This can again be explained by the fact that the same VENC was used for all three velocity components, while the velocities along the *z* axis are considerably higher than those along the *x* and *y* axes (the energy of $w_{\mathrm {mri}}$ was 29.76 dB higher than that of $u_{\mathrm {mri}}$, and 27.09 dB higher than that of $v_{\mathrm {mri}}$), and is a direct (and desirable) consequence of the denoising properties of the proposed approach, as previously discussed.

**Table 3:** Signal-to-error ratio (in dB) between the in vivo experiment results from each of the three CFD approaches—pure CFD (labeled “CFD”); CFD driven by one PC-MRI velocity component (labeled “CFD + 1D”); and CFD driven by all three PC-MRI velocity components (labeled “CFD + 3D”), relative to the MRI-measured velocity field

	CFD	CFD + 1D	CFD + 3D
${\mathrm {SER}}_{u}$	0.51	0.50 ($\approx$)	0.75 ($\uparrow$)
${\mathrm {SER}}_{v}$	−1.51	$-$1.87 ($\downarrow$)	$-$0.92 ($\uparrow$)
${\mathrm {SER}}_{w}$	4.74	14.53 ($\uparrow \uparrow$)	10.74 ($\uparrow$)
${\mathrm {SER}}_{\vec {\nu }}$	4.13	4.89 ($\uparrow$)	6.28 ($\uparrow \uparrow$)

SER values were calculated according to Eqs. (21)–(24)

## Discussion

The proposed methodology uses the three-dimensional SIMPLER algorithm with Cartesian uniform meshes to perform blood flow simulations under the influence of three-dimensional MRI-measured velocity profiles. The combined MRI–CFD methodology attempts to correct the MRI-measured flow field, forcing it to satisfy the fluid mechanics equations. The choice of the SIMPER algorithm and Cartesian discretization was performed in order to facilitate implementation of the algorithm. We showed that the proposed technique provides better agreement with the PC-MRI measurements than pure CFD simulations. We also showed that this MRI-guided CFD approach can be used as a means of reducing noise in the PC-MRI measurements. It can also be used to reduce computation time: when all three MRI-measured velocity components are used to guide the CFD simulations, only a few iterations are required for convergence, i.e., for finding the flow field that is the most similar to the PC-MRI measurements (in the least squares sense) while satisfying the fluid mechanics equations.

We believe that the proposed method can also be used as a technique for reducing scan time. The proposed methodology allows the use of only part of the velocity measurements obtained with MRI to guide computational solutions by appropriately choosing the $\Gamma$ matrices in Eqs. (12)–(14). In this paper, we used identical grids for MRI and CFD. Therefore, the $\Gamma$ matrices were square with size $N\times N$. In each of the following suggested approaches, at least one of the $\Gamma _u$, $\Gamma _v$ or $\Gamma _w$ matrices may be of size $M\times N$, with $M<N$. Possible approaches for reducing scan time include: (1) acquiring the MRI data with reduced spatial resolution, and using the MRI-guided CFD simulation to improve the spatial resolution; (2) measuring portion(s) of the volume (e.g., carotid inlet and outlets) with full spatial resolution, while measuring the rest of the volume with reduced spatial resolution; (3) acquiring only a few slices along the bifurcation, and using the MRI-guided CFD simulation to fill in the gaps; (4) measuring one or two velocity components with full spatial resolution, while measuring the other component(s) with reduced spatial resolution; (5) measuring one or two velocity components across the entire volume, while measuring the other component(s) for only a few slices; and (6) any combination of these approaches. We plan to explore these ideas in future studies.

It is well known that blood is a non-Newtonian fluid, therefore its viscosity is not uniform. While there exists many constitutive models and studies in the literature regarding the non-Newtonian rheological properties of blood [43, 53, 54], no gold-standard constitutive model exists, and the assumption of constant whole blood viscosity is common practice [13, 16, 33, 37, 55, 56]. Therefore, we used the Newtonian blood flow model (constant whole blood viscosity), which greatly simplified the implementation.

It is often desirable to estimate the wall shear rate at the carotid bifurcation. Determining wall shear rates from CFD simulation results would require using highly refined meshes around the neighborhood of the vessel wall. Our implementation of the SIMPLER algorithm does not allow using spatially-varying grid spacing, and the mesh is uniform all over the integration domain. Using a very fine grid over the entire integration domain is possible, in principle. However, this would drastically increase the computational complexity, and could make the proposed methodology impractical in a clinical environment, for example.

Finally, the proposed approach does not take the effects of vessel wall elasticity into consideration. While the pulsatile carotid flow phantom we used has rigid tube walls, the human carotid vessel wall is generally elastic. While there exists blood flow CFD simulation methods that incorporate elastic wall effects [57, 58], the assumption of rigid vessel walls is another common practice [13, 16, 33, 37, 55]. The SIMPLER algorithm implemented in this work uses a Cartesian uniform mesh, and does not allow the use of the elastic wall models.

Both wall shear stress calculations and elastic vessel walls could be properly addressed with an implementation using finite elements—which would allow an easier adaptation of the mesh near the wall and also allow simulating the effects of fluid–structure interaction. Despite the fact that the implementation of a finite-element solver would be substantially more complex than our implementation, all the proposed methodology described in this proof of concept paper can still be applied in the same fashion, since the problem of solving the set of differential equations in a finite element discretization is replaced by a sparse system of linear equations similar to the ones obtained in this work.

In future works, this MRI-guided CFD methodology for unsteady flow will be implemented on FreeFem++,^1^ a partial differential equation solver capable of solving the Navier–Stokes equations using finite elements [59]. FreeFem++ allows access to the linear systems that can be modified in order to make use of the principles introduced here. With this approach, we expect more general, higher-quality solutions, allowing the calculation of biomarkers, such as wall shear stress, since FreeFem++ can handle different types of triangular finite elements, large varieties of linear system solvers, and automatic mesh adaptation. Note that there are other free software that could be used to reproduce this methodology, such as openFOAM,^2^ which uses finite volume discretization. Both FreeFem++ and openFOAM allow modifications of the linear systems, and have CFD solvers already implemented, which could facilitate the implementation of the methodology proposed in this study [40, 59].

All the assumptions and simplifications disscussed above (Newtonian blood flow, imprecise geometry, non-compliant walls) contribute non-linearly to the differences observed between CFD solutions and MRI-measured velocity fields. Using the MRI-measured velocity field to constrain the CFD solution indirectly addresses these simplifications, and provides a more realistic CFD solution. However, we are still unable to clearly identify the main factors responsible for the disagreement between MRI-measured and CFD-computed velocity fields. Nevertheless, an implementation using a more robust solver, as proposed above, could improve on these limitations and further reduce the gap between MRI-measured and CFD-computed results.

## Conclusion

We have proposed a framework for obtaining flow field estimates that are influenced by both PC-MRI measurements and a fluid physics model. The results showed that the proposed technique provides better agreement with the PC-MRI measurements than pure CFD simulations, and has reduced computation time (faster convergence). MRI-guided CFD can be used to correct the MRI-measured flow field, forcing it to satisfy the fluid mechanics equations. It can also be used as a means of reducing noise in the PC-MRI measurements, and has potential as a method for reducing scan time.

The proposed framework offers a general approach to in vivo blood flow assessment, that is complementary to improvements in PC-MRI acquisition and reconstruction techniques, and can be applied to the study and diagnosis of a broad range of cardiovascular flow mapping applications.

## Footnotes

1 http://www.freefem.org.

2 http://www.openfoam.org.
